# Molecular Biomarkers for Contemporary Therapies in Hormone Receptor-Positive Breast Cancer

**DOI:** 10.3390/genes12020285

**Published:** 2021-02-17

**Authors:** Allegra Freelander, Lauren J. Brown, Andrew Parker, Davendra Segara, Neil Portman, Brandon Lau, Elgene Lim

**Affiliations:** 1 Garvan Institute of Medical Research, Darlinghurst, NSW 2010, Australia; a.freelander@garvan.org.au (A.F.); Lauren.Brown5@health.nsw.gov.au (L.J.B.); andrew.parker@svha.org.au (A.P.); d.segara@garvan.org.au (D.S.); n.portman@garvan.org.au (N.P.); brandon.bz.lau@gmail.com (B.L.); 2 Faculty of Medicine, St Vincent’s Clinical School, UNSW Sydney, Darlinghurst, NSW 2010, Australia; 3 St Vincent’s Hospital, Darlinghurst, NSW 2010, Australia

**Keywords:** biomarkers, breast cancer, estrogen receptor, prognostic, predictive, pharmacodynamic

## Abstract

Systemic treatment of hormone receptor-positive (HR+) breast cancer is undergoing a renaissance, with a number of targeted therapies including CDK4/6, mTOR, and PI3K inhibitors now approved for use in combination with endocrine therapies. The increased use of targeted therapies has changed the natural history of HR+ breast cancers, with the emergence of new escape mechanisms leading to the inevitable progression of disease in patients with advanced cancers. The identification of new predictive and pharmacodynamic biomarkers to current standard-of-care therapies and discovery of new therapies is an evolving and urgent clinical challenge in this setting. While traditional, routinely measured biomarkers such as estrogen receptors (ERs), progesterone receptors (PRs), and human epidermal growth factor receptor 2 (HER2) still represent the best prognostic and predictive biomarkers for HR+ breast cancer, a significant proportion of patients either do not respond to endocrine therapy or develop endocrine resistant disease. Genomic tests have emerged as a useful adjunct prognostication tool and guide the addition of chemotherapy to endocrine therapy. In the treatment-resistant setting, mutational profiling has been used to identify *ESR1*, *PIK3CA*, and *AKT* mutations as predictive molecular biomarkers to newer therapies. Additionally, pharmacodynamic biomarkers are being increasingly used and considered in the metastatic setting. In this review, we summarise the current state-of-the-art therapies; prognostic, predictive, and pharmacodynamic molecular biomarkers; and how these are impacted by emerging therapies for HR+ breast cancer.

## 1. Introduction

Breast cancer is now the most common cancer and most common cause of cancer-related death in women. With an expected mortality rate of approximately 30%, the burden for patients, families, and society is high. The majority of these deaths are attributed to metastatic disease, which has often developed resistance to standard-of-care treatments and become clinically intractable for current therapeutic strategies.

Breast cancer classification and management is guided by its immunohistochemical (IHC) and molecular subtype, where prognostic information can be determined and response to therapy predicted [1,2]. Tissue-based biomarkers, including the expression of estrogen receptor (ER), progesterone receptor (PR), and human epidermal growth factor receptor 2 (HER2), have been integral in subtyping of tumours, prognostication, and choice of systemic therapies. 

Approximately 70–80% of all breast cancers are hormone receptor-positive (HR+) and molecularly classified as luminal A or luminal B [2,3,4,5]. Currently, endocrine therapies such as selective ER modulators (SERMs) and aromatase inhibitors (AIs) remain the standard of care adjuvant systemic therapy for primary HR+ breast cancer (Table 1). Extended endocrine therapy for 10 years has been shown to decrease the risk of recurrence relative to 5 years of therapy [6,7]. In addition, ovarian suppression improves survival when added to adjuvant endocrine therapy in premenopausal women who have also received chemotherapy [8]. Adjuvant bisphosphonates have also been shown to decrease recurrence risk in post-menopausal patients [9]. With the routine use of adjuvant endocrine therapy changing the natural history of HR+ breast cancer, the biological features of breast cancer recurrence are sometimes different from primary tumours. For example, metastatic ER+ tumours have a higher rate of *ESR1* mutations (range 12–56%) compared to primary breast cancers (0.4%, The Cancer Genome Atlas (TCGA), https://www.cancer.gov/tcga (accessed on30 December 2020).) [10,11,12,13,14].

In the metastatic setting, endocrine therapy, including selective ER degraders (SERDs), are used in combination with cyclin-dependent kinases 4 and 6 inhibitors (CDK4/6i), and represent the current gold standard for early lines of therapy [15,16,17,18,19]. These therapies have improved survival end-points, with a median progression-free survival (PFS) of up 28 months and a median overall survival (OS) of up to 40 months in the first-line setting reported to date (Table 2). Therapies that target the phosphatidylinositol 3-kinase (PI4K)-mammalian target of rapamycin (mTOR) such as everolimus (an mTOR inhibitor) and PIK3Cα inhibitors have also shown to be effective in combination with endocrine therapy in metastatic disease [20,21,22,23] (Table 1). 

## 2. Molecular Biomarkers in Breast Cancer

In the validation and discussion of clinically relevant biomarkers, it is important to differentiate between prognostic, predictive, and pharmacodynamic biomarkers. Prognostic markers assess the biology of the disease, and can help determine the probable outcome as either favourable or unfavourable [36]. Predictive biomarkers aid in determining which therapies will be efficacious for individual patients. Finally, biomarkers that show that a biological response has occurred in an individual who has been exposed to a medical product are said to be a pharmacodynamic/response biomarker [36]. While there are distinctions between these different classes of biomarkers, it should be noted that they often overlap. For example, an ER+ status indicates a favourable prognosis, and predicts response to endocrine therapies (Table 3). 

Although traditional biomarkers have proven to be valuable in breast cancer, it is a highly heterogeneous and polyclonal disease where tissue-based biomarkers cannot fully capture the spatial and temporal evolution of a tumour as it is exposed to multiple therapies. Additionally, sequential tissue-based biopsies to test for biomarkers present a logistical challenge due to its invasive nature and sampling error. The detection of molecular biomarkers and gene signatures through alternative methods such as circulating tumour DNA (ctDNA) analyses thus complement these traditional methods, as they allow for non-invasive assessment of the disease, as well as for the study of multiple time points throughout treatment, allowing for the study of tumour evolution and treatment response [37]. 

## 3. Histological Biomarkers in ER + Breast Cancer

### 3.1. Tumour Grade

Tumour morphology can provide information on its behaviour and response to therapy. Histologically, breast tumours can be classified according to grade using the Nottingham Grading System (NGS). Multiple studies have identified NGS as a valuable and prognostic factor independent of tumour size and the number of positive lymph nodes [38,67,68]. Tumour grade is strongly associated with relapse-free survival (RFS), breast cancer-specific survival (BCSS), and disease-free survival (DFS) [69]. Thus, histological determination of tumour grade is a robust marker in breast cancer. 

### 3.2. Pathological Prognostic Stage

Since being defined by expert consensus with the American Joint Committee on Cancer (AJCC), the pathological prognostic stage group is a modification of the anatomical TNM (tumour, nodes, metastases) staging system that includes histological biomarker grade and ER/PR/HER2 receptor status [39]. The AJCC TNM staging system has used tumour size, extent of nodal involvement, and presence or absence of metastases as a ubiquitous tool across all cancers to understand disease behaviour, provide prognostic information, and guide treatment decisions since 1977 [70]. In validation studies, these histological features can divide each anatomical stage category into distinct risk groups (each comparison *p* < 0.05) [71] and can provide meaningful new information with up to 31% and 28% upstaged and downstaged, respectively, when compared to anatomical information alone [72].

### 3.3. Estrogen Receptor

Expression of ER is a hallmark of hormone-dependent breast tumour growth and a predictive and prognostic marker in breast cancer. ER’s usefulness as a biomarker stems from its dependence on binding to estrogenic ligands, such as 17β-estradiol, for the regulation and activation of its canonical signalling pathway. Estradiol is one of the most important mitogens in ER+ breast cancer, whereby ER–estradiol transcriptional complexes initiate signalling pathways that promote cell survival and growth [73]. CyclinD1 is such a transcriptional target of ER, which binds to CDK4 and -6, and phosphorylates the retinoblastoma protein (Rb). Phosphorylated Rb releases E2F transcription factors and promotes entry into S phase. Additionally, ligand-bound ER induces c-Myc expression where subsequent CDK4 upregulation initiates S-phase.

According to American Society of Clinical Oncology/College of American Pathologists (ASCO/CAP) guidelines, breast tumour sections are considered ER+ where 1 to 100% of tumour nuclei stain positive for ER by IHC analysis. Additionally, samples are deemed ER-low where only 1 to 10% of nuclei are positive [74]. 

ER+ breast cancers have a better prognosis compared with ER-negative breast cancer [40]. However, up to 30% of patients will relapse in spite of adjuvant endocrine therapy, and the late recurrence risk continues many years following initial diagnosis [75,76]. Interestingly, quantitative measurements of ER load by IHC in stage 1-3 early breast cancer have not been found to provide predictive or prognostic information [41]. Moreover, patients with a higher ER load did not benefit better from endocrine therapy than patients with a low ER load. This indicates that ER should only be considered at the qualitative level to be valuable as a biomarker. ER is also a strong predictor for effectiveness of anti-estrogen therapies; 5 years of adjuvant tamoxifen was found to be associated with 12.9–14.9% absolute decrease in 15-year cancer recurrence for women with ER+ cancers, but there was no apparent effect in those with ER-poor disease (RR 0.97, 95% confidence interval 0.88–1.07) [24]. It remains the defining predictive biomarker for all forms of endocrine-based therapy in the early and advanced stages of ER+ breast cancer.

### 3.4. Progesterone Receptor

PR is both an ER-induced gene target and a modulator of ER behaviour, and is expressed in more than 50% of ER+ tumours [77]. In the presence of PR agonists, such as progesterone (P4) or progestins, PR associates with ER and directs ER chromatin binding events within breast cancer cells, resulting in a gene expression signature that is associated with a good clinical outcome [78]. Preclinical data have also uncovered that estradiol-mediated growth of ER+ tumour models were inhibited by PR agonists in combination with tamoxifen [78], and clinical trials are currently underway to evaluate this.

PR status is determined through immunohistochemical analysis of either core biopsy or surgical specimens. Guidelines set by the ASCO/CAP cut off PR+ cases as ≥1% PR+ tumour cells [44]. Thus, tumours that stain for <1% positive cell nuclei are considered PR-negative. A cut off value of 20% is also recommended by the European Society for Medical Oncology (ESMO) to distinguish between high and low PR expression [79].

PR expression is a valuable prognostic biomarker in breast cancer. Low and negative expression of PR in ER+ tumours is associated with a more aggressive and proliferative disease, resulting in poorer prognosis and clinical outcome [44,80]. However, its value as a predictive biomarker is controversial. Conflicting studies have found PR either a useful marker of response to endocrine therapy, or that no additional predictive value is observed when compared to ER [24,43,81]. In a meta-analysis of randomised trials of adjuvant tamoxifen, PR was not found to be a predictive biomarker of response to tamoxifen [24], and quantitative studies of PR load has found no clear evidence as a predictive marker [41]. In contrast, the TransConfirm trial demonstrated a better PFS (hazard ratio (HR) 0.59, CI 0.38–0.91, *p* = 0.016) in patients with a tumour with a PR Allred score of ≥6, indicating that PR load may be a predictive factor in SERD therapy [82]. 

### 3.5. Androgen Receptor

The androgen receptor (AR) is expressed in up to 80% of all breast cancers; however, it is more commonly co-expressed in primary and metastatic ER+ cancers (90% and 75%, respectively) [83,84,85]. The use of AR as an independent prognostic biomarker could be of benefit. The effects of AR signalling have been observed in the clinic, where ER+/AR+ tumours are typically smaller, well differentiated, and a prognostic factor for longer disease-free survival [45]. The magnitude of AR positivity by IHC analysis is also correlated with better survival in retrospective analyses of ER+ cohorts [83]. This has been investigated in multiple studies, with higher levels of AR conferring a survival advantage [83,86,87]. Additionally, expression of AR is positively correlated with PR expression [78,88]. Recent preclinical studies have demonstrated that selective AR modulators (SARMs) have promising anti-proliferative activity in ER+ breast cancer models [89]. However, there is currently no clinical role of AR as a predictive biomarker.

### 3.6. Human Epidermal Receptor 2 (HER2)

The human epidermal receptor 2 protein (HER2/ErbB-2) belongs to the ErbB family, which consists of plasma membrane-bound receptor tyrosine kinases. Dimerisation of HER2 results in phosphorylation of tyrosine residues in the cytoplasmic domain and initiates signalling in the Ras/MAPK, PI3K/Akt, STAT pathways, thus promoting cell proliferation, migration, adhesion, and survival [90]. When overexpressed, multiple HER2 heterodimers are formed and an enhanced responsiveness to growth factors and proliferation is observed [90]. 

The detection of HER2 overexpression by IHC, or amplification by fluorescence in situ hybridisation (FISH), is both a prognostic and predictive indicator. Approximately 15–20% of all breast cancers test positive for the HER2 gene [77,91,92]. Typically, HER2-amplified tumours are associated with a higher pathological grade and more extensive forms of ductal carcinoma in situ (DCIS) [93,94]. Moreover, multivariate analyses show a correlation of poor clinical outcome with HER2 gene amplification, indicating that HER2 is a prognostic factor [91]. However, HER2-amplified tumours exhibit sensitivity to anti-HER2 therapies, such as trastuzumab (a monoclonal HER2 antibody) or lapatinib (a small molecule HER2 inhibitor) [46,95]. Thus, HER2 is also a predictive biomarker, where the determination of HER2 status is an important factor in the stratification of patients to an effective therapeutic strategy.

While *ERBB-2^mut^* are relatively uncommon in breast cancers, they are enriched in metastatic tumours (approximately 1.5% vs. 5% in early and advanced disease, respectively) [96,97]. Hotspot mutations including NP_001005862:p.Asp769His, p.Val777Leuc, p.Arg896Cys, p.Ser310Phe, and p.Leu755_Thr759del in the extracellular, transmembrane, and kinase domains result in constitutive ligand-independent HER2 signalling [97]. Importantly, two clinical trials have demonstrated clinical efficacy of neratinib (a small molecule pan-HER2 inhibitor) in patients with *ERBB-2^mut^* breast cancer [59,60].

### 3.7. Ki67

Ki67 is a marker of cell proliferation and is expressed in the nucleolus of cells [98]. While it is widely used in histopathology, a major limitation is inter-reporter variability [99]. International guidelines have been established for its measurement and use in breast cancer [100]. The St Gallen consensus cut-off values for Ki67 as measured by IHC analysis are ≥20% for “high”-level expression and <20% for low-level expression. Together with tumour grade and PR expression, it is endorsed to assist in differentiation of luminal A and B subtypes of breast cancer [101,102], and often used to as an additional variable in adjuvant therapy decisions.

Ki67 expression is associated with tumour grade, mortality [103], disease-free survival, and distant recurrence-free survival [104]. A ≥20% increase in Ki67 expression following neoadjuvant endocrine therapy is associated with worse disease-free survival and OS compared to stable or declining Ki67 expression [105]. A Ki67 of ≤2.7% has been used as an end-point and termed complete cell cycle arrest in a number of clinical studies of preoperative combination endocrine therapy and CDK4/6i clinical trials [106,107,108]. This was originally described in earlier neoadjuvant endocrine therapy studies to determine tumour features in terms of outcome prediction, where a Ki67 of ≤2.7% following therapy was found to be associated with improved survival outcomes [109,110].

A pharmacodynamic response in Ki67 to endocrine therapy +/− CDK4/6i was also observed in the Poetic trial, where Ki67 following 2 weeks of therapy improved prediction over baseline Ki67 alone [48,49]. Furthermore, tumours that did not exhibit a Ki67 response following preoperative endocrine therapy were enriched for genomic drivers of endocrine resistance, such as *FGFR1* and *CCND1* amplification, as well as intrachromosomal *ESR1* fusions and enhanced E2F-mediated transcription [111,112]. These findings further validate the detection of Ki67 at multiple time points as a pharmacodynamic prognostic marker.

### 3.8. PD-L1 and TILs

Programmed death ligand 1 (PD-L1; also CD274) is a transmembrane protein expressed on normal tissues that acts on its receptor PD-1 to modulate and suppress the immune system [113]. Over-expression of PD-L1 in cancer is thought to co-opt this mechanism, leading to immune system evasion, one of the modern hallmarks of cancer [114]. While its expression by IHC analysis can be utilised as a biomarker in breast cancer, there is no consensus about assay choice; cut-off; or measuring tumour cells, immune cells, or both [115]. One study using modified Histo-score with a positive cut-off ≥ 100 found 23% of 650 evaluable breast cases to be PD-L1+, as well as an association with worse prognosis, both in the overall cohort and the HER2-negative luminal B subset [116]. Other studies found PD-L1+ in 20–56% across all breast subtypes depending on definition, with either no association or association with better prognosis; notably, the ER+ subset was not analysed separately in these studies [50,117,118].

Regarding the predictive utility of PD-L1, while it was associated with improved response to atezolizumab immunotherapy in the phase 3 randomised study IMpassion130 [119], its role in ER+ cancer is less clear. One study defined PD-L1 as a continuous variable using automated quantitative analysis in 94 patients receiving neoadjuvant chemotherapy—PD-L1 was associated with pathological complete response (PCR), and this was also seen in the ER+ subgroup [51]. PD-L1 appears to have merit as both a prognostic and predictive biomarker, although there are methodological challenges that must be overcome before it enters widespread use in the clinic.

Tumour-infiltrating lymphocytes (TILs) are another potential biomarker reflective of the interplay between the immune system and breast cancer cells. The International TILs Working Group 2014 recommends standardised methodology for assessing stromal TILs by IHC analysis [120], and this approach has minimal inter-operator variability [121]. Increasing TILs in the 1466 ER+/HER2-negative cases analysed within a pooled cohort treated with neoadjuvant chemotherapy across six clinical trials found association with worse prognosis (HR 1.10 for shorter OS per 10% increase in TILs, 95% confidence interval 1.02–1.19) but was predictive of pathological complete response (PCR; rate 6%, 11%, and 28% in low, intermediate, and high TILs, respectively) [52]. Regarding response to endocrine therapy, 106 patients receiving 4 months of neoadjuvant letrozole for ER+ breast cancer in the phase 2 Danish Breast Cancer Group study found each 10% increase in TILs was associated with decreased chance of PCR (odds ratio (OR) 0.71, 95% confidence interval 0.53–0.96) [53]. While TILs appear to be both prognostic and predictive in ER+ breast cancer, validation in prospective clinical trials is needed.

## 4. Genomic and Genetic Biomarkers in ER+ Breast Cancer

Genome analysis has led to the identification of novel biomarkers with both prognostic and predictive implications. These genomic biomarkers, DNA or RNA characteristics within breast cancer cells, highlight the importance of gene testing in the future of precision cancer therapeutics. Several genetic factors have been identified as drivers of breast cancer, including mutations of *PIK3CA, TP53, ESR1*, and *MYC* amplification [122] (TCGA, https://www.cancer.gov/tcga (accessed on 30 December 2020).). Furthermore, these gene alterations, particularly *ESR1* and *PIK3CA*, have been identified as predictive biomarkers of therapeutic response [123]. As multigene sequencing can be applied to tumour tissue. Specific gene testing for pathogenic genetic variants can also be assessed through copy number analysis (e.g., multiplex ligation-dependent probe amplification, MLPA) or Sanger sequencing without the need for whole-genome analysis.

Evolving ctDNA technology may allow for fast, routine, and dynamic molecular assessment of tumour alterations without the need for tissue biopsy [124]. While tissue biopsies are a mainstay in the genomic characterisation of a breast tumour, there is a significant discordance between primary and metastatic tumours, requiring multiple samples to be taken throughout treatment [125]. Tissue biopsies are an invasive and uncomfortable procedure that may not yield enough tumour for analysis, due to either sampling error or inaccessible sites [126] (235, 198). ctDNA analysis overcomes these issues, as multiple, non-invasive samples can be taken with minimal discomfort [127]. Importantly, analysis of tumour DNA is possible in cases where metastatic lesions are located in inaccessible sites, and both spatial and temporal heterogeneity of the primary and metastatic tumour can be examined [128,129].

### 4.1. Genomic Assays

Multiple commercially available genomic assays are currently approved for use in HR+ breast cancer. Oncotype Dx and MammaPrint remain the only tests with prospective clinical trial evidence [54,55]. Other genomic assays to assess breast cancer recurrence risk include Breast Cancer Index [130], EndoPredict [131], and Prosigna [132]. However, their data are limited to retrospective evaluations of clinical trial populations [133], and currently there remains insufficient data to recommend one genomic biomarker assay over another [133]. 

These tests can be analysed as both prognostic biomarker tools regarding the risk of breast cancer recurrence, as well as to provide predictive information about the benefit of adjuvant chemotherapy or endocrine therapy [134]. Oncotype Dx is a 21-gene assay assessing 16 breast cancer-related genes and 5 reference genes to report a breast cancer recurrence score of low, intermediate, or high risk, which is both prognostic for recurrence at 10 years and predictive of benefit from chemotherapy [54]. Low scores (<10) reflect that anti-estrogen therapy alone is sufficient therapy, whereas high scores (>26) reflect poor prognosis and typically predict benefit from chemotherapy in addition to endocrine therapy. In TailoRx, a prospective clinical trial of HR+/HER2−, lymph node-negative breast cancers with an intermediate Oncotype Dx recurrence score (11–25) were randomly allotted to receive either chemo-endocrine therapy or endocrine therapy alone [54]. Initial results indicated that endocrine therapy was non-inferior to chemo-endocrine therapy, further establishing this genomic risk score as a predictive biomarker vis-à-vis adjuvant chemotherapy benefit. However, its predictive value can be refined by including other patient and disease risk factors. Subsequent analyses support the benefit of chemotherapy in women under the age of 50 years with scores in the higher end of the intermediate risk group (21–25). Those with scores at the lower end of the intermediate risk group (16–20) will benefit from chemotherapy if their clinical risk is also high (e.g., high grade and large tumour size) [135].

A 70-gene expression profile test, MammaPrint, is an alternative assay that determines a low or high genomic risk assessment in patients with node-negative or 1–3 positive lymph node disease [55]. Whilst the Ki67 marker is a large component of the Oncotyope Dx weighted score, this is not among the genes included in the MammaPrint assessment. The prospective Mindact trial assessing the clinical applications of MammaPrint demonstrated that adjuvant endocrine therapy was noninferior to chemoendocrine therapy in women with high clinical risk and low genomic risk. Thus, the MammaPrint assay can be utilised as a predictive biomarker to identify patients with low genomic risk who can safely forgo adjuvant chemotherapy. 

### 4.2. BRCA1/2 Mutation

*BRCA1* and *BRCA2* are tumour suppressor genes that produce the key enzymatic pathways for homologous recombination-mediated repair of double-stranded DNA breaks. Typically, pathogenic *BRCA1/2^mut^* are detected through germline testing of normal whole blood or saliva DNA, or somatic testing of tumour tissue [136,137]. It has been demonstrated that patients with germline *BRCA1/2^mut^* did not portend worse breast cancer-specific outcomes compared to non-carriers; however, this study was not analysed in accordance with ER status [138]. A more recent study suggested that patients with germline *BRCA1/2^mut^* ER+ tumours had a 2.3 times higher risk of disease recurrence and 3.4 times higher risk of breast cancer-related deaths compared to non-carriers [61].

Mutations in these genes cause chromosomal instability, and chromosome breaks are thought to be predictive biomarkers for response to poly(ADP-ribose) polymerase (PARP) inhibitors or platinum chemotherapy [139]. ER+ breast cancer accounts for approximately 22% and 77% of invasive breast cancers in germline *BRCA1^mut^* and *BRCA2^mut^* carriers, respectively [140]. 

Whilst BRCA1 and −2 proteins play a significant role in homologous recombination, BRCA1 is also involved in base and nucleotide excision repair. The key enzyme for the DNA excision repair pathway is PARP-1. PARP inhibitors halt the repair of single strand breaks, leading to DNA replication fork collapse or formation of double-strand DNA breaks that require homologous recombination to restore. In vitro studies suggest that in cells deficient of BRCA1 or −2 that are unable to perform the process of homologous recombination, PARP inhibition leads to “selective cytotoxicity”, or cell death [139]. 

The investigators of the phase 3 OlympiAD study concluded the use of the oral PARP inhibitor olaparib demonstrates efficacy in patients with a germline *BRCA1/2^mut^* (loss of BRCA function) in both ER+ and triple-negative tumours [30]. In patients with somatic *BRCA1/2^mut^*, with maintenance of normal BRCA protein functionality, PARP inhibitors have minimal effect [139]. In patients with BRCA-related breast cancer, higher response rates have been observed in those treated with platinum over taxane chemotherapy [141]. This is explained by the formation of DNA cross-links by platinum salts, which would normally be repaired by homologous recombination dependant on the *BRCA1* and −2 genes. While *BRCA1/2^mut^* are emerging as predictive molecular biomarkers for platinum-based chemotherapy for triple-negative breast cancer, its role in HR+ disease is less established.

### 4.3. PI3K/AKT/mTOR Pathway Alterations

PI3K acts to phosphorylate proteins and lipid molecules and are overactive in a large proportion of breast cancers. PI3K triggers downstream activation of AKT (also known as protein kinase B) and mTOR, thus forming the PI3K/AKT/mTOR signalling pathway leading to cell growth, metabolism, proliferation, and survival [142]. A total of 40% of patients with ER+ breast cancer have activating *PIK3CA^mut^,* representing the most common genomic alteration in breast cancer [143] (TCGA, https://www.cancer.gov/tcga (accessed on 30 December 2020).). Hotspot *PIK3CA^mut^* includes NP_006209.2.4:p.Glu542Lys and p.Glu545Lys in exon 10 (helical domain), and p.His1047Tyr in exon 21 (kinase domain) [144]. The dysregulation of this pathway initiates bidirectional crosstalk and subsequent modulation of the ER to continue ER-dependent growth [145]. Moreover, activating mutations in AKT phosphorylates CDK4/6 and CDK2 inhibitors p21 and p27, moving them away from their cyclin/CDK targets and further promoting cell cycle progression [146,147]. Loss of PTEN results in increased PI3K activity and may lead to clinical resistance to endocrine therapy and has been associated with shorter relapse-free survival following tamoxifen treatment [148]. Eventually these pathways will begin to act as ER-independent drivers of growth, thus leading to endocrine resistance. 

*PIK3CA^mut^* act as a prognostic biomarker and are associated with improved survival outcomes in patients with early-stage HR+/HER2− breast cancer (Table 4) [144,149]. In contrast, while the frequency of *PIK3CA^mut^* does not differ significantly between early stage and metastatic HR+/HER2− breast cancer, patients with metastatic *PIK3CA^mut^* disease have been shown to have a poorer outcome and resistance to chemotherapy compared with *PIK3CA^wt^* tumours [150].

Both the Solar1 and Sandpiper trials demonstrated that patients with *PI3KCA^mut^* disease have increased response rates and survival with the use of alpelisib (a PI3Kα-specific inhibitor) and endocrine therapy. This benefit was only seen in patients with *PIK3CA^mut^* tumours or *PI3KCA^mut^* ctDNA, demonstrating its utility as a predictive biomarker (median PFS 11 vs. 5.7 months, HR 0.65, *p* < 0.0001; median OS 39.3 vs. 31.4 months, HR 0.86, *p* = 0.15) [20]. The Sandpiper trial tested the addition of taselisib (a β-sparing PI3K inhibitor) to fulvestrant with similar results in patients with *PI3KCA^mut^* tumours (median PFS 7.4 vs. 5.4 months, HR 0.70, *p* = 0.0037) [151]. The overall survival data remain immature. 

Neoadjuvant trials assessing the utility of alpelisib and taselisib showed varied results. The Neo-Orb trial assessing the addition of alpelisib did not appear to improve objective response or pathological complete response rates when used with letrozole [152]. In contrast, the Lorelai trial of taselisib plus letrozole was predictive of response in the *PIK3CA^mut^* population OR 56% vs. 38% OR 2.03 (95% CI 1.06–3.88) [153]. The Opportune trial assessing pictilisib (a pan-PI3K inhibitors) did not support *PIK3CA^mut^* as predictive of efficacy in the neoadjuvant setting [154]. 

Whilst mTOR inhibitors have been shown to be efficacious in conjunction with endocrine therapy in HR+ metastatic breast cancer, there are currently no predictive biomarkers identified. The Bolero-2 trial demonstrated that the addition of the mTOR inhibitor everolimus to exemestane increased the median PFS (6.9 vs. 2.8 months, HR 0.43, *p* < 0.001) in patients who had previously progressed on aromatase inhibitors [21]. Pre-clinical studies have demonstrated that tumours with *PIK3CA^mut^* have increased sensitivity to everolimus, but this has not been confirmed in clinical studies [155]. Further mutational analysis of tumours of patients from the Bolero-2 and TamRad (tamoxifen +/− everolimus) studies did not demonstrate a relationship between everolimus efficacy with *PIK3CA^mut^* and *PTEN* status [156]. 

More recently, capiversertib (an AKT inhibitor), alone and in combination with fulvestrant, demonstrated clinical activity in a phase 1 study of patients with the activating mutation *AKT1^E17K^* endocrine-resistant metastatic ER+ breast cancer, which represent approximately 7% of this subtype, offering further evidence that genomic alterations in this signalling pathway may be effectively therapeutically targeted [58].

### 4.4. ESR1 Mutations

Mutations within the ERα encoding gene, *ESR1,* are frequently acquired in HR+ breast cancer and were only recently recognised as a clinically relevant entity. These mutations are thought to be mediated through clinical acquired resistance to prior aromatase inhibitor therapy. *ESR1^mut^* are gain-of-function mutations that are largely clustered within a “hotspot”, the ligand-binding domain (AF2) of the ER [11,157]. Within this hotspot, missense mutations located in residues NP_000116.2:p.Tyr537Ser, p.Tyr537Asp, p.Ser537Cys, and p.Asp538Gly are the most common, while p.Leu536Glu, p.Leu536Arg, p.Pro535His, p.Ser463Pro, and p.Val534Glu have also been described [13,158]. These mutations have been described to contribute to tumour progression and endocrine therapy resistance [159,160,161]. Functionally, these mutations change the conformation of the ligand-binding domain and render the ER protein stably active in the agonist mode [159]. Consequently, co-activators are able to bind to ER in the absence of a ligand and initiate transcription independently of estrogen. Thus, these cells exhibit reduced sensitivity to endocrine therapies. In contrast to the TCGA study of primary breast cancers, where the frequency of *ESR1^mut^* was <1% (https://www.cancer.gov/tcga (accessed on 30 December 2020).), *ESR1^mut^* detected by analyses of circulating free DNA (cfDNA) have found a higher proportion (28–55%) in metastatic ER+ breast cancers previously treated with SERD or AI therapy [12,57,162,163]. 

*ESR1^mut^* strongly correlate with endocrine therapy resistance and provide important prognostic and predictive information. *ESR1^mut^* cancers are resistant to estrogen deprivation and are therefore less responsive to endocrine therapies [13]. Furthermore, clinical studies have associated these mutations with poorer outcomes compared with *ESR1^wt^* tumours [12]. Fulvestrant, a first-generation SERD, has been shown to be relatively more effective than aromatase inhibitors in the presence of *ESR1^mut^*; however, resistance is still frequent [57]. Currently, second-generation SERDs that have preclinical activity in fulvestrant-resistant and *ESR1^mut^* preclinical models are undergoing early phase trials, representing the most promising treatment strategy in *ESR1^mut^* breast cancer.

Exploratory results from the phase 3 Pada-1 trial suggest that *ESR1^mut^* as measured in DNA may have utility as a pharmacodynamic biomarker [164]. Among the subgroup of 33 patients with baseline *ESR1^mut^* detected at baseline, “clearance” of this mutation in response to therapy with aromatase inhibitor and palbociclib was associated with a prolonged PFS compared with patients who had a persistent *ESR1^mut^*. More prospective trials are needed to validate the use of *ESR1^mut^* as a biomarker in this way.

### 4.5. ER Activation Gene Signature

While molecular markers have transformed our understanding of breast cancer, and provide vital prognostic and predictive information in the clinic, there is a lack of established biomarkers that predict response to SERDs [82]. Moreover, there is a need for a molecular marker or gene set to predict response or resistance to endocrine therapy in metastatic ER+ disease. A gene set of estradiol-induced and -repressed genes whereby an ER activity score was determined in the cell line and in patient-derived xenograft (PDX) models treated with a SERD has been developed [56]. Interestingly, different ER ligands drive a distinct transcriptional response in both cell line and PDX models, whereby greater transcriptional suppression of ER correlated with stronger anti-tumour activity [56]. This has the potential to provide valuable predictive information to guide treatment decisions. 

In addition, gene expression and pathway analyses from Affymetrix microarrays of locally advanced or metastatic ER+ tumours in the TransConfirm trial uncovered unique ER-mediated gene signatures that conferred decreased response to fulvestrant [82]. Expression of a set of 37 genes was found to be independently associated with PFS, where upregulation of 27 of these genes were known to have functions in the regulation of the ER transcriptional network (*FOXA1* [165], *TFAP2C* [166], *SP1* [167]) [82]. These genes were also associated with breast cancer biology and clinical outcome (e.g., *TFAP2C, BMPR1A* [168], *ARRDC3* [169]). Interestingly many genes in this set had not yet been reported in breast cancer biology, but have functional roles in tumorigenesis or response to treatment in other cancer types (e.g., *USP5* [170]) [82]. 

### 4.6. Cell Cycle Molecules (Rb, Cyclin D1, and FAT1) 

The combination of CDK4/6i with endocrine therapy is now standard-of-care first-line therapy for advanced breast cancer. However, despite impressive benefits to PFS, not everyone responds to therapy and most patients relapse over time. Currently, only the presence of ER and wild-type retinoblastoma protein (Rb) have been established as clinical biomarkers to predict response to this combination.

CDK4/6i prevent phosphorylation of Rb, thus allowing it to deactivate the E2F transcription factors that coordinate the early stages of cell cycle progression [92]. Several preclinical studies have implicated loss of function of Rb as a driver of CDK4/6i resistance [171,172]. Analyses of ctDNA detected *RB1^mut^* in 1.7% of patients across three randomised trials of ribociclib, with an enrichment in polyclonal *RB1^mut^* detected in samples collected after CDK4/6i treatment [62,171,173]. Interestingly, the addition of ribociclib with endocrine therapy showed no increase in PFS for these patients, indicating that *RB1^mut^* may confer cross-resistance to endocrine therapy and CDK4/6i [64]. A panel of 87 genes that correspond to loss of Rb function, even in the presence of an intact *RB1* gene, has also been identified. This panel (RBsig) was able to differentiate between palbociclib-sensitive and -resistant cell lines [63]. While these results indicate that *RB1* may be a predictive and potentially pharmacodynamic biomarker, further validation is required to determine its clinical use.

Cyclin E:CDK2 phosphorylates Rb downstream of CDK4/6 in late G1 of the cell cycle. Amplification of the *CCNE1* gene, which encodes cyclin E1, and loss of *RB1* is associated with the development of resistance to CDK4/6i, with the ratio of *CCNE1/RB1* identified as a marker of palbociclib resistance [174]. This pattern was also associated with poor OS in the Metabric dataset. Amplification of *CCNE1* predicted resistance to palbociclib in the NeoPalAna clinical trial dataset [106], and overexpression of *CCNE1* was associated with palbociclib resistance in retrospective RNA analyses of both the Paloma-3 and Preoperative Palbociclib (Pop) trials [175]. In analyses of palbociclib-resistant MCF7 cells with amplification of *CCNE1*, the ablation of CDK2 and cyclin E1 re-sensitised cells to CDK4/6i [172], suggesting a mechanistic role for cyclin E1:CDK2 in CDK4/6i resistance. Upregulation of either cyclin E or CDK2 may therefore compensate for CDK4/6 inhibition by offering alternative pathways for Rb deactivation and constitute promising pharmacodynamic biomarkers.

Loss of function of the protein FAT1, a negative regulator of the pro-survival HIPPO pathway [176], has been identified as a potential driver of CDK4/6i resistance [64]. Loss of FAT1 results in an accumulation of HIPPO pathway transcription factors that induce overexpression of CDK6, which in turn increases the growth inhibitory concentration of CDK4/6i, allowing tumours to proliferate. In a clinical study, targeted sequencing of tumour and patient-matched normal DNA samples, from FFPE or peripheral blood, on 348 patients treated with palbociclib, ribociclib, or abemaciclib correlated *FAT1* alterations with reduced PFS. Cases with biallelic *FAT1* inactivation showed a drastic reduction in PFS (2.4 months), compared to patients with *FAT1^wt^* tumours (10.1 months) [64]. *FAT1^mut^* were detected in only 6% patients with metastatic disease and therefore may have a limited role as a predictive biomarker in question. The consequence of FAT1 loss—upregulation of CDK6—may prove more useful. Dysregulation of other parts of the HIPPO pathway similarly induce upregulation of CDK6 and consequent resistance to CDK4/6i [64]. Upregulation of CDK6 as a result of gene amplification has been reported in abemaciclib-resistant breast cancer cell lines in which knockdown of CDK6 restored sensitivity to abemaciclib [177]. *FAT1^mut^* may therefore be just one example of a broader phenomenon that does convey predictive power. 

## 5. Imaging Biomarkers

Functional nuclear medicine scans have recently been developed specifically for ER+ breast cancer, specifically 16α-[^18^F]fluoro-17β-estradiol (FES) positron emission tomography (PET) [178]. In FES-PET, the positron-emitting radioisotope ^18^F is bound to 17β-estradiol and delivered to tissues overexpressing ER; the subsequent avidity of imaged tissues is reported as a standardised uptake value (SUV) [179]. Increased uptake on FES-PET is associated with histological ER expression with agreement comparable to standard in vitro assays [180], and thus would be predictive of response to endocrine therapy. Higher FES-PET uptake was found to be a positive prognostic biomarker for 90 patients with metastatic ER+ breast cancer treated with endocrine therapy: PFS of 7.9 months (95% confidence interval 5.6 to 11.8 months) and 3.3 months (95% confidence interval 1.4 to not evaluable) for FES-PET uptake above and below the average SUV, respectively [65]. This study demonstrated that the combination of imaging markers that can assess both likelihood of response to endocrine therapy and disease aggressiveness with traditional FDG-PET improved the ability to predict PFS over either modality alone. Similarly, changes in FES-PET uptake may be prognostic—in a small study of 22 patients with ER+ metastatic breast cancer, patients achieving PFS greater than 12 months had greater change in maximum decrease in SUV: 91.0 ± 12.0% vs. 20.7 ± 16.2% [181].

Perhaps more tantalising is the use of FES-PET as a pharmacodynamic biomarker for novel SERDs in clinical trials. Earlier work found that ER availability by FES-PET fell in patients receiving tamoxifen or fulvestrant, but less so with aromatase inhibitors (54% versus 15% fall in maximum SUV; *p* < 0.001) [182]. The oral SERD elacestrant led to reduced ER availability by FES-PET measure in 16 patients on a phase 1b study, although the magnitude of SUV fall did not correlate with objective response rate or PFS [66]. This may be due to a threshold effect—a phase 1 dose escalation trial of another novel SERD SAR439859 demonstrated a decrease in FES uptake (>87%) in all patients achieving plasma concentration >100 ng/mL, contributing to the selection of the recommended phase 2 dose in clinical trials [183]. The utility of FES-PET in this way is promising for early phase studies, but further validation is required before it can be used outside of clinical trials.

## 6. Discussion

The use of traditional tissue-based biomarkers such as ER and PR in the prognostication and prediction of response to therapy is well established. However, with increasing understanding of molecular pathways in breast cancer and the emergence of novel targeted therapies, there is a need for new biomarkers to be established. Recently, the development of multigene and mutational profiles discussed in this review have opened avenues of research into new therapeutic targets and potential biomarkers. Furthermore, with the decreasing cost and increasing availability of genomic and panel testing for breast cancer-specific mutations, these profiling methods may prove to be a cost-effective strategy to guide therapy in the clinic. The combination of emerging therapies effective against specific genomic aberrations and ctDNA technology as an accessible alternative to invasive tumour biopsies points to a future where genomic biomarkers could be used for personalised therapy, as well as being a means of performing serial, almost real-time, assessment of the evolutionary mutational landscape in response to treatment. 

Another promising development is the identification of pharmacodynamic biomarkers, such as 2-week Ki67 response, ER activation gene signatures, *ESR1^mut^*, *PIK3CA^mut^*, cell cycle components, and FES-PET imaging. In the metastatic setting, these markers have the potential to personalise approaches to treatment and predict the development of resistance. However, despite the identification of cell cycle components such as Rb and cyclin E1 as pharmacodynamic markers in CDK4/6i resistance, collective data are not concordant and there is currently no clinical use. A coordinated and streamlined approach to biomarker in parallel with drug development, utilising cutting-edge platforms such as genomic testing and imaging modalities, while expensive, would in the long term improve both the cost-effectiveness and development timeline of new treatment strategies. Finally, rigorous comparison of these emerging markers to those already established is essential, along with strategies to integrate them into existing screening in order to further improve outcomes.

## Figures and Tables

**Table 1:** Summary of current treatment strategies for hormone receptor-positive (HR+) breast cancer.

Treatment	Comparator	Measure of Effect	Reference
**Early Stage Breast Cancer**			
Endocrine Therapy in Postmenopausal Women			
5 year tamoxifen	Placebo	15 year breast cancer mortality RR 0.70 (95% CI 0.64–0.75), *p* < 0.00001	[24]
5 year total therapy (tamoxifen → AI)	5 year tamoxifen	10 year breast cancer mortality RR 0.85 (95% CI 0.75–0.96), *p* = 0.015	[9]
5 year AI	5 year tamoxifen	10 year breast cancer mortality RR 0.85 (95% CI 0.75–0.96), *p* = 0.009	[9]
5 year total therapy (AI → tamoxifen)	5 year AI	8 year DFS HR 1.06 (95% CI 0.91–1.23), *p* = 0.48	[25]
10 year extended therapy (tamoxifen)	5 year tamoxifen	Benefit highest after 10 year, e.g., 10–14 year breast cancer mortality RR 0.71 (95% CI 0.58–0.88), *p* = 0.01	[6]
10 year extended therapy (AI)	5 year AI → 5 year placebo	5 year DFS HR 0.66 (95% CI 0.48–0.91), *p* = 0.01	[26]
Endocrine Therapy in Premenopausal Women			
5 year tamoxifen	Placebo	15 year breast cancer mortality RR 0.70 (95% CI 0.64–0.75), *p* < 0.00001	[24]
5 year tamoxifen + LHRH agonist	5 year tamoxifen	Benefit in high-risk, e.g., 8 year DFS HR 0.76 (95% CI 0.60–0.97) in those receiving adjuvant chemotherapy	[8]
5 year AI + LHRH agonist	5 year tamoxifen	Benefit in high-risk, e.g., 8 year DFS HR 0.68 (95% CI 0.53–0.88) in those receiving adjuvant chemotherapy	[8]
Chemotherapy and Other Systemic Therapy			
Anthracycline + taxane regimen	Anthracycline w/o taxane regimen	8 y breast cancer mortality RR 0.86 (95% CI 0.79–0.93)	[27]
Bisphosphonate	No bisphosphonate	In postmenopausal women, OS HR 0.77 (95% CI 0.66-0.90), *p* = 0.001; no benefit seen in premenopausal women	[28]
**Advanced Breast Cancer**			
Endocrine + Targeted Therapy in Postmenopausal Women			
AI + CDK4/6 inhibitor	AI + placebo	See Table 2	
Fulvestrant + CDK4/6 inhibitor	Fulvestrant + placebo	See Table 2	
Fulvestrant + alpelisib	Fulvestrant + placebo	In patients with *PIK3CA^mut^*, median PFS 11.0 vs. 5.7 month, HR 0.65 (95% CI 0.50–0.85), *p* < 0.001	[20]
Exemestane + everolimus	Exemestane + placebo	Median PFS 6.9 vs. 2.8 month, HR 0.43 (95% CI 0.35–0.54), *p* < 0.001	[21]
Endocrine + Targeted Therapy in Premenopausal Women			
As a general principle, many targeted strategies with AI or fulvestrant backbone above can be combined with LHRH agonist for premenopausal women			
Tamoxifen + ribociclib	Tamoxifen + placebo	See Table 2	
Chemotherapy and Other Systemic Therapy			
Sequential mono-chemotherapy	NA	e.g., taxane, anthracycline, capecitabine, eribulin, vinorelbine, gemcitabine	[29]
Combination chemotherapy	NA	Consider in patients with visceral crisis	[29]
PARP inhibitor	Physician’s choice chemotherapy	In patients with germline *BRCA1/2^mut^*, median PFS 7.0 vs. 4.2 mth, HR 0.58 (95% CI 0.43–0.80), *p* < 0.001	[30]
Denosumab	Bisphosphonate *	Skeletal-related event RR 0.78 (95% CI 0.72–0.85), *p* < 0.001	[28]

AI: aromatase inhibitor; AC: doxorubicin (Adriamycin) and cyclophosphamide; BRCA1/2: breast cancer gene 1/2; CDK4/6: cyclin-dependent kinase 4/6; HR: hazard ratio; LHRH: luteinising hormone-releasing hormone; OR: odds ratio; RR: risk ratio; DFS: disease-free survival; PFS: progression-free survival; OS: overall survival; mth: month. * For patients with bone metastases.

**Table 2:** Seminal trials of CDK4/6i in advanced breast cancer.

	First-Line				Second-Line			*n*th-Line
**Trial**	Paloma 2	Monarch 3	Monaleesa 2	Monaleesa 7	Paloma 3	Monarch 2	Monaleesa 3	Monarch 1
**Drug**	Palbociclib	Abemaciclib	Ribociclib	Ribociclib	Palbociclib	Abemaciclib	Ribociclib	Abemaciclib
**Population (pre- or post-menopausal)**	Post-M	Post-M	Post-M	Pre-M	Pre- and Post-M	Post-M	Post-M	Post-M
**Endocrine therapy**	AI	AI	AI	OS + AI/Tam	Ful 500 + OS (in Pre-M)	Ful 250	Ful 500	Nil
**Phase**	III	III	III	III	III	III	III	II
**N**	666	493	668	660	521	669	484	132
**Randomisation**	2:1	2:1	2:1	2:1	2:1	2:1	2:1	Single arm
**PFS/TTP (mth)**	24.8 vs. 14.5	28.2 vs. 14.8	25.8 vs. 16.0	23.8 vs. 13.0	9.5 vs. 4.6	16.4 vs. 9.3	20.5 vs. 12.8	6
**HR**	0.58; *p* < 0.001	0.54; *p* < 0.001	0.57; *p* < 0.001	0.55; *p* < 0.001	0.46; *p* < 0.0001	0.55; *p* < 0.001	0.59; *p* < 0.001	NA
**OS (mth)**	NR	NR	NR	Not reached vs. 40.9	34.9 vs. 28.0	46.7 vs. 37.3	Not reached vs. 40.0	17.7
**HR**	-	-	-	0.71; *p* = 0.01	0.81; *p* = 0.09	0.76; *p* = 0.014	0.72; *p* = 0.05	-
**Reference**	[31]	[32]	[19]	[17]	[16]	[33]	[34]	[35]

AI: aromatase inhibitor; OS: ovarian suppression; Tam: tamoxifen; Ful: fulvestrant; NR: not reported; PFS: progression-free survival; TTP: time to progression; 95% CI: 95% confidence interval; HR: hazard ratio; OS: overall survival.

**Table 3:** Biomarkers in HR+ breast cancer.

Biomarker	Prognostic	Predictive	Pharmacodynamic	Reference
**Histopathology**				
Tumour grade	Y	N	N	[38]
Pathological prognostic stage	Y	N	N	[39]
ER	Y	Y. to ET-based therapy	N	[9,40,41,42]
PR	Y	N	N	[43,44]
AR	N	N	N	[45]
HER2	Y	Y. to HER2-targeted therapy	N	[46]
Ki67	Y	N	N	[47]
Ki67 response to 2 weeks of preoperative ET	Y	N	Y	[48,49]
PDL1 and TILs	Y. to TILs only	Y. to immunotherapy	N	[50,51,52,53]
**Genomic**				
Multigene tests	Y	Y. to chemotherapy *	N	[54,55]
ER activation gene signature	N	Y. to SERDs	N	[56]
*ESR1^mut^*	Y	Y. to SERDs	Y	[12,57]
*PIK3CA* ^mut^	Y	Y. to alpelisib	N	[20]
*AKT^mut^*	N	Y. to capivarsertib	N	[58]
*ERBB2^mut^*	N	Y. to HER2-targeted therapy	N	[59,60]
*BRCA1/2^mut^*	Y	Y. to PARPi	N	[30,61]
Cell cycle molecules	N	Y. to CDK4/6i	N	[62,63,64]
**Imaging**				
FDG-PET	Y	N	Y	[65]
FES-PET	Y	Y. to SERDS	Y	[65,66]

ET: endocrine therapy; ER: estrogen receptor; PR: progesterone receptor; AR: androgen receptor; PDL1: programmed death ligand 1; TIL: tumour-infiltrating lymphocytes; SERD: selective estrogen receptor downregulator; ESR1: estrogen receptor 1; PIK3CA: phosphatidylinositol-4,5-bisphosphate 3-kinase catalytic subunit α; BRCA1/2: breast cancer gene 1/2; CDK4/6: cyclin-dependent kinase 4/6; PARPi: poly(ADP-ribose) polymerase inhibitor; FDG-PET: fluorodeoxyglucose positron emission tomography; FES-PET: 16α-[^18^F]fluoro-17β-estradiol positron emission tomography. Y represents clinically validated biomarkers to date. * Oncotype and Mammaprint only.

**Table 4:** Seminal trials of PI3Ki and mTORi + endocrine therapy in advanced breast cancer.

	First-/Second-Line	Second-Line	First-/Second-Line	Second-Line
**Trial**	TamRad	Bolero	Solar 1	Sandpiper
**Drug**	Everolimus	Everolimus	Alpelisib	Taselisib
**Population (pre- or post-menopausal)**	Post-M	Post-M	Post-M	Post-M
**Endocrine therapy**	Tam	NSAI	Ful	Ful
**Phase**	III	III	III	III
**N**	111	724	572	516
**Randomisation**	1:1	2:1	1:1	2:1
**PFS/TTP (mth)**	8.6 vs. 4.5	10.6 vs. 4.1	11.0 vs. 5.7	7.4 vs. 5.4
**HR**	0.54; *p* = 0.002	0.36; *p* < 0.001	0.65; *p* < 0.001	0.70; *p* = 0.004
**OS (mth)**	Not reached vs. 32.9	31 vs. 26.6	NR	NR
**HR**	0.45; *p* = 0.007	0.89; *p* = 0.14	-	-
**Reference**	[23]	[21,22]	[20]	[151]

AI: aromatase inhibitor; NSAI: non-steroidal aromatase inhibitor; Tam: tamoxifen; Ful: fulvestrant; NR: not reported; PFS: progression-free survival; TTP: time to progression; HR: hazard ratio; OS: overall survival; mth: months.

## Data Availability

Not applicable.
